# Medical and Philosophical News

**Published:** 1781-03

**Authors:** 


M. Alphonfo le Roy, phyfician at Paris, in a cafe of putrid fever, after applying blifters, and employing antifeptics and cordials to no purpofe, had recourfe to phofphorus; two grains of it were diffolved in a fpoonful of linfeed oil, mixed with with two ounces of water. The patient took a ſpoonful of this mixture every hour. In the account of this caſe, publiſhed in the *Gazette de Santé*, we are told, that during eight days previous to the exhibition of this remedy, the patient (a young man of twenty-four) had voided his urine involuntarily, that his pulſe was ſunk, the pupils of his eyes dilated, and his face and extremities cold and inſenſible. He began to take the phoſphorus at night; the next morning his pulſe was ſtronger, and he was in every reſpect better; the day following he voided his fæces and urine naturally. Six of theſe mixtures were given in the ſpace of ſeven days, and the patient gradually recovered.—This is not the firſt inſtance of the internal uſe of phoſphorus. Kunckel is ſaid to have preſcribed it in the form of pills, and ſome German phyſicians have gone ſo far as to give twelve grains of it for a doſe.*

